# Oral Health Barriers for African American Caregivers of Autistic Children

**DOI:** 10.3390/ijerph192417067

**Published:** 2022-12-19

**Authors:** Dominique H. Como, Lucía I. Floríndez-Cox, Leah I. Stein Duker, Sharon A. Cermak

**Affiliations:** 1 Mrs. T.H. Chan Division of Occupational Science and Occupational Therapy, Herman Ostrow School of Dentistry, University of Southern California, Los Angeles, CA 90089, USA; 2 Nursing Research and Performance Improvement Department, Cedars-Sinai Medical Center, Los Angeles, CA 90048, USA

**Keywords:** oral health, autism, African American, barriers, health equity, children

## Abstract

The most persistent oral health disparities in the United States impact children from racial and ethnic minoritized groups and children diagnosed as autistic. This paper aims to describe barriers to oral care as depicted by Black/African American (B/AA) parents of autistic children to further explore how and why oral health disparities persist in this population. A purposeful sample of eleven caregivers of autistic children, ages 4 to 14 years, who identified as B/AA were interviewed twice for approximately 60–90 min each. Thematic analysis utilizing a narrative approach was employed. Three themes emerged from the data concerning the barriers that affect oral health experiences: (a) difficulty in maintaining good oral health practices, (b) challenges with access to care and resources, and (c) poor patient-provider relationships. Due to the limited research that examines the intersection of autism, B/AA culture, and oral health practices, this study provides a rich picture of the barriers families face when obtaining oral care. Many families raised issues that other parents of autistic children also identified. B/AA caregivers have demonstrated that despite their own negative dental experiences, they understand the value of good oral care practices and are willing to pursue oral care for their children.

## 1. Introduction

Health Equity is described as the fair opportunity to achieve and attain the highest level of health potential and not be disadvantaged because of social position or unjust systemic barriers [1,2,3,4]. Indeed, health inequities can lead to increased risks for the type, duration, severity, and impact of disease and disability. While acknowledging health disparities has led to increased attention and focus on certain health issues, health inequities persist, especially in populations that are medically underserved, minoritized, or have barriers to accessing quality care [5]. Disparities are not the result of a single cause; they are often the result of several complex, dynamic, and intersectional reasons [6]. One of the most common unmet health needs for children in the United States is oral health [3,5,7,8].

The most persistent oral health disparities impact racial and ethnic minoritized groups; African Americans/Blacks, Hispanics/Latinos, and American Indians/Native Alaskans have the worst overall oral health outcomes, even after controlling for socioeconomic status [3,9]. Poor oral health has been linked to heart disease, stroke, and diabetic complications, and overwhelmingly impacts African Americans; targeting oral health is therefore critical to improving the overall health of these populations [10]. The health of African Americans is affected by increased risk factors and higher incidence, morbidity, and mortality rates of many chronic diseases and conditions when compared with Caucasians [11,12]. Previous research has identified several structural, sociocultural, and familial factors as impacting the ability of African Americans to utilize oral care services [9]. Oral health practices (i.e., in-home care, preventative dental visits) for African American parents may be influenced by limited knowledge about risk factors, cultural beliefs, and norms via preferences for cultural remedies, biases about preventative health, or mistrust of health care professionals [13,14,15,16,17].

Another population experiencing oral health related inequity is those with special health care needs [18,19]. For example, autistic children are at greater risk of experiencing oral health disparities than the general population [20]. [Throughout the paper we use identity-first language (“autistic child”) because it is the preference of many in the autism community [21,22]. However, we use person-first language (“child with autism”) in accordance with matching and respecting the ways the participants described themselves or their children in the interviews]. This elevated risk may result from difficulties in implementing oral care practices due to differences in communication, sensory processing sensitivities, distressed behaviors, restrictive eating habits, and dental fear and anxiety [8,22,23,24,25]. For example, multiple studies report that only half of the autistic children brush their teeth twice daily as recommended, and up to 70% of parents of autistic children report that toothbrushing, even once a day, is difficult [26,27]. This challenge may be exacerbated by the sensory demands of the activity, such as the taste and texture of toothpaste or the tactile sensation of the toothbrush bristles [22,28]. In addition, caregivers of autistic children report limited access to dental practitioners who are both trained and willing to treat their child [27,29].

African American autistic children may face unique and largely unresearched oral challenges. Intersectional identities can lead to a complex, interconnected web of privileges and disadvantages. Unfortunately, there is not always an equal distribution of advantages; sometimes, the outcome can result from additive disparity. For example, African American children are often diagnosed as autistic at a later age than their peers and thus often do not receive services promptly [30]. This may delay their ability to engage in preventative treatments (e.g., desensitization), exacerbating the factors contributing to African American children’s poor oral health.

Several qualitative studies have explored oral health for autistic children, with caregivers sharing that sensory over-sensitivity, lack of autism awareness of dental professionals, and challenges in parent-provider partnerships, among others, are barriers to good oral health [14,25,31,32,33,34]. For example, in a study with male autistic youth, parents described how difficult it was to find a dentist [31]. Similarly, parental expertise has been identified as essential in multiple qualitative studies [14,25,34]. However, despite the persistence of oral health disparities, limited research exists regarding oral care for African American autistic children. Therefore, this paper aims to describe barriers to oral care as depicted by African American parents of autistic children to further explore how and why oral health disparities persist in this population.

## 2. Materials and Methods

The data reported are part of a larger convergent parallel mixed-method study designed to understand the factors contributing to the persistence of oral health disparities for African American families. This study was approved by the University of Southern California Institutional Review Board (HS-19-00995).

### 2.1. Recruitment

This study was part of a larger study with 125 African American participants who completed an online survey about oral care knowledge, attitudes, and behaviors [24]. Respondents who indicated that they had an autistic child were asked to indicate if they were interested in sharing more about their lived experiences around oral health. Survey participants who stated that they were willing to be contacted regarding participation in the qualitative interview component were contacted. A purposeful sampling strategy was utilized to select the participants and attempts to capture the heterogeneity across the population were considered. For example, efforts were made to include participants with children across the age range (i.e., 4–7 years, 8–11 years, and 12–14 years), gender, and socioeconomic status (see Table 1). Parents who expressed interest were screened to ensure that variation remained amongst those selected to participate. Inclusion criteria included parent-report of autism diagnosis, parent participation in the online survey, a child between the ages 4 to 14 years, and English speaking.

### 2.2. Procedures

A doctoral student and a qualitative methodology expert developed a semi-structured narrative interview guide based on the information obtained from a literature review to provide depth and richness to understanding the factors that inform oral health practices of African American families with autistic children. Through this interview, we gathered information related to: (1) background (parent/caregiver, child), (2) health services (i.e., health coverage, provider availability, quality of care), (3) physical environment (i.e., geography, food access) (4) biological factors (i.e., fear and anxiety, sensory sensitivities), (5) individual factors (i.e., health literacy, parenting style) and (6) social environment (i.e., social supports, stigma). We also collected basic demographic information from all participants.

This study originally included only in-person interviews; however, due to the COVID-19 pandemic, an updated protocol was approved and implemented to allow for the interviews to be conducted utilizing Zoom, which was found to be an effective method [35]. After completing the informed consent process, caregivers were interviewed twice for approximately 60–90 min each via the Zoom platform. All interviews were conducted by a doctoral student trained in qualitative research and interview techniques. Interviews were audio-recorded via Zoom and transcribed verbatim by a professional transcription service.

### 2.3. Data Analysis

Thematic analysis utilizing a narrative approach was employed. The thematic narrative analysis focuses on identifying information that can aid in exploring the issue [36]. Data analysis was an immersive, ongoing, and iterative process [37]. Each transcription was read and reread to identify emergent themes and create initial codes. A preliminary list of codes was inductively developed from the data by one member of the research team. Then, two research study team members independently analyzed the data set utilizing a qualitative data analysis computer software (NVivo 12) for coding. The entire research team reviewed themes and codes for congruency. Discrepancies were resolved through discussion until a consensus was reached.

## 3. Results

### 3.1. Participants 

The participants included eleven caregivers of autistic children, ages 4 to 14 years, who identified as Black/African American. As seen in Table 1, all interviewees were mothers, with one father also participating. The majority of children represented were boys, consistent with ASD prevalence [38]. Participants were distributed across the child’s age range of 4 to 14 years and from different regions of the United States. Most parents completed at least some college, while income varied widely across participating families. Based on the Social Responsiveness Scale -Second Edition, approximately half the children were reported by their parents to have mild to moderate autism symptoms, with the other half reported as severe [39].

### 3.2. Themes

Three themes emerged from the data concerning the barriers that affect the oral health experiences of African American families of autistic children: (a) difficulty in maintaining good oral health practices, (b) challenges with access to care and resources, and (c) poor patient-provider relationships.

#### 3.2.1. Difficulty in Maintaining Good Oral Health Practices

In this theme, parents described reasons why it was challenging to maintain good oral health for their child, this ranged from their own experiences to their child’s sensory needs. In addition, they offered the suggestion of professional support to aid in the process.

Parental Experiences. All parents described their own negative dental experiences as children and adolescents and how those occurrences left them with varying levels of dental fear and anxiety as adults. For example, one parent shared:

My mom wasn’t able to go back [to the treatment room] with me one day, and they were trying to do something to my mouth, and I was scared. Instead of them going to get my mom to calm me down, they literally put me in a chokehold and shot me in the mouth with the needle…I’ve been scared ever since.

Despite these past traumatic dental experiences, which impacted some participants’ willingness to seek dental care even as adults, not one parent described their fears and anxiety as preventing them from seeking oral care for their child. Most parents conveyed the same sentiment as the following mother, who described how her own negative experiences with poor oral health served as the impetus for how she cared for her child. The parent said:

I guess it’s because I don’t want them to end up like me. Like be so young with cavities and missing teeth. And I know my momma didn’t take us [to the dentist] a lot. So, I want to take them a lot, especially my son, because he [does not] talk. And if he was to have a cavity, I mean if he was to be in pain from a cavity, I wouldn’t know because he doesn’t talk. He’ll just cry.

Communication. In addition to situating her own experiences of suboptimal dental care and reduced access to care as a child, this parent described how having an autistic child amplified her awareness of needing to take different actions for her child, and speak up about things. She also relayed the difficulty of providing care for her autistic son as his limited communication abilities impede knowing when he is in pain. Other parents supported this sentiment and acknowledged that it would be challenging to know what might be affecting their autistic child without regular dental visits as children’s nonverbal communication may not be adequate for pointing the caregiver to an area of concern or pain.

Sensory-related challenges with oral care. In addition to communication, parents also described how sensory sensitivities impacted oral health practices at home and in the dental clinic. One caregiver shared that utilizing an electric toothbrush was challenging for her child due to their sensory needs, “Maybe it’s too much—the sound, the vibration. Keeping the toothpaste on the brush, and it’s splattering on different parts of his mouth, I think probably might not be fun for him”. So, despite the recommendation from the dental professional to use an electric toothbrush instead of a manual toothbrush to aid in-home oral care practices, the sensory stimuli of the electric toothbrush proved to be too much for the child to tolerate, resulting in decreased frequency of toothbrushing for this child, as his caregiver was unable and unwilling to force him to continue using it.

The sensory demands of a dental clinic visit also can result in distressed behaviors and lead to barriers for the child, caregiver, and practitioner. One parent described the sensitivities their child experiences in the dental clinic, stating:

In the beginning, I think, when it comes to the vacuum suction, she doesn’t like that. So, the dentist lowers it [the suction power]. But if it’s just a loud sound, like the drilling, she doesn’t like it. When she was getting her cavities filled, I think the drilling kind of caused an issue for her, or the noise. And she started to get upset, had a tantrum, started getting aggressive. She started kicking. Her father and my dad actually went to help.

The caregiver went on to describe how the behaviors demonstrated by their child in the clinic led to subsequent treatments requiring general anesthesia for completion, which caused the caregiver to be apprehensive about seeking care. Another parent shared how their child’s behaviors in the clinic due to their sensory sensitivities led to a traumatic experience, causing them to refrain from further treatment for several years. She stated,

They wanted to strap her in, which I didn’t want them to do. I don’t know. I think I wrapped the blanket around her once, and then when I left the room, they tried to strap her in. It just as so traumatic. It’s like imagine being strapped in and it’s already sensory overload and whatnot…So, maybe we didn’t go for three or four years [after that].

Oral care routines in the home. Additionally, many parents shared that getting their autistic child to incorporate the recommended oral care practices into their daily routines usually required additional familial assistance and/or professional teaching. One parent explained their child’s oral care practices this way:

What I try to do, because he tries to be independent, is I let him brush his teeth first. But then when I look at his teeth, and I don’t like how he brushes them, I’m kinda OCD, I’ll grab a toothbrush and start doing it over…Then he gets mad and bites down on the toothbrush, and it’s kind of like I’m battling back and forth with it. It is hard sometimes…But I try to brush his teeth every day.

Another participant described her children’s oral care practices and how the siblings support each other, through the use of media (a Cocomelon song) to help them brush, this way:

Basically…when he sees [his siblings] in [the bathroom], he’ll come in, and they’ll sing, ‘Teeth, teeth, you have to brush your teeth.’ And then he’ll say, ‘No, no, no. I don’t want to brush my teeth’. Then they’ll say, ‘Please. Please. Brushing is good for you’. And he’ll say, ‘Yeah, yeah, yeah. I like that. Woo’. Then he’ll let them brush them. But if I say it, he’s like, ‘No’.

Another participant described the challenge with her son, “I try to get my autistic son to brush at least twice a day, but it’s a struggle. You’ve got to hold him down and put him—lock him under your legs just to brush his teeth in the morning”.

Another parent shared,

When you have a child on the spectrum, it’s overwhelming… I would say depending where you[r] kid is on the spectrum, and how much support you need to give that kid, it may affect what things you have to do for your kid. Like for me, I’m not making any excuses, but my kid needs everything. He needs help in everything that he does. He’s learning. He has challenges every day as far as skills.

Professional support. Other caregivers used the help of professionals to address behaviors or sensory needs of their child that made oral care activities difficult. The following participant spoke about how the occupational therapist [OT] adapted their session to include oral practices into the treatment plan when the parent was having trouble getting their autistic child to use the toothbrush, saying:

…she [the OT] would cut his session down and work ten minutes of teaching him how to brush his teeth. That did help because it was like once he seen that she stepped in to help, then it made it a little bit easier for us to get his teeth brushed because she would keep a toothbrush specifically for my son. Right before his session was over, they would go across the hall, and she would take him in the back and show him how to brush his teeth. It kind of made the situation a little bit better.

Another caregiver described how their behavioral therapists helped them to develop an in-home oral care routine.

So, obviously, initially it was like teaching him how to brush his teeth. So, home-based behavioral therapy helped with that. They helped with all that, and the flossing. And then we also use a fluoride rinse. So, he does that in the morning when he wakes up, and then he does that at night. He flosses usually once a day. He probably should do more than that. But, yeah, I think the dentist wanted him to do more than that.

#### 3.2.2. Challenges with Access to Care and Resources

Parents reported challenges in this theme related to access to resources as a barrier to oral health, especially when intersected with the cost and amount of care required to maintain other aspects of health care for their autistic child.

Financial Barriers. One parent described the stress she feels when contemplating going to the dentist because of the amount of services needed and potentially high cost of the treatment for her autistic child:

I don’t want to go. I need to go. That’s honest. And I will go, but I don’t want to go. I don’t want to sit there and it’s like, ‘Okay. Are you going to have to pull teeth?’ Just issues that I have. It’s like, ‘Okay. Well, you’re going to give me this laundry list of things or things that I can’t afford.’… So, you know, that’s just stressful for me, to deal with the reality of the expense—along with, ‘How do we get this done along with everything else?’

She was not the only caregiver to voice concerns about the cost and amount of dental services indicated as necessary for their autistic child. Multiple parents explained that it is not just the high cost of the services, but the emotional stress they feel about the uncertainty of those costs and how they will afford them in addition to all the other services and resources they need to provide for their autistic children. One parent equated the pressure she feels going to the dentist with the same feeling she experiences taking her car to the mechanic; the comparison being that she is not an expert in the field, so she has to implicitly trust the information being provided to her as truthful and necessary, saying:

I’m always a little skeptical when we go to the dentist for my child. What are they going to find? It’s kind of like when you bring your car in to get a checkup. Okay, they’re going to find a $1200 issue, or a $300 issue? You know what I’m saying?

Dental insurance issues. Several participants described challenges related to their dental insurance coverage and how that translated to inequitable treatment and access difficulties. Primarily, dental insurance allows caregivers to seek out providers for oral care services, and have some cost-savings for subsidized treatment choices. However, these choices are not infinite, especially for children who have state-sponsored public insurance which is provided to low-income families. One parent shared the difficulty of finding a dentist in their local community, “…it’s hard finding someone who accepts our insurance that’s within a reasonable distance from our house”.

Moreover, several parents explained feelings of disparity in the quality of treatment they received based on the type of their insurance (e.g., public, private). As this participant describes, it is not only the lack of options but also the feeling that the quality is just passable when insurance coverage is state-sponsored:

I truly believe that it depends on your medical insurance coverage. That definitely affects how you’re treated as far as medical or health…But when they find out, for me and my family, we’ve had [state-sponsored insurance] for a while. And you know, [state-sponsored insurance] doesn’t cover a lot of things. And it definitely makes a difference as far as what type of service you get… If you have like [state sponsored insurance], which is the standard or the government insurance, everything has so many limitations on what, you get the bare minimum, you know.

Another participant echoed a similar experience of blatant unjust treatment, being segregated to different waiting rooms based on their insurance type. They said:

They treat you differently based on whether you can pay or what your insurance is. Yes, you’re treated differently. At this one dentist office, I forgot, that we went to when my kids were younger, they actually had a—he had us enter separately. And I don’t know whether it was considered a bias, per se. But he had his office divided…the people that had [state sponsored insurance] went to this particular section, and then the ones that had the other insurance went to the other section.

Community deficit. Participants also disclosed their perception of limited or lacking community resources, especially for families with autistic children. Several parents described their physical environment as under-resourced, and seemed to feel excluded from existing programming due to lack of promotion in their community. Moreover, there was a noted lack of programming for autistic children. One participant shared that finding community resources can be complex, often requiring a great deal of effort and research, saying:

There’s not enough of anything. You really have to dig for stuff and find your own resources. If there is a community event or something for kids, you don’t know about it because there’s no advertising. I’m like hours on the internet always searching for things and sometimes you find one thing, you find something else, but definitely not in the community. There’s definitely not enough information for [families of children with autism]. You could have a great program up the corner and not know about it because it’s not—it’s word of mouth or not advertised right or whatnot.

Noted in this example is the realization that a program could be occurring in the neighborhood, but community members may never know about it because advertising often is relegated to word of mouth. Another parent shared a similar response, highlighting their disappointment with the advertising of programs intended for members of the community, which often go unutilized due to lack of awareness, stating:

My friend, who lives a block from me, didn’t even know [a specific program] existed, so that’s the kind of thing. It was our place right down the block from me and she didn’t know it existed and it’s like, ‘We pass these things all the time’, and if you’re not aware—if they’re not trying to get things out in the community—there’s a program in the corner and I’m not even sure what they do, but I know that I see kids in there all the time. I’ll see kids of other races and it’s just like, ‘How do you even know about this? I’m the one who lives in the neighborhood’. I’m just like…nobody who lives around here knows about it, so there’s definitely a lack of promotion of resources within the community.

The following participant expressed her dissatisfaction with the lack of resources available to the community-at-large, and is particularly lacking for the autistic community, and how that has negatively impacted her autistic child’s progress, saying:

I think the biggest thing for African Americans in the autism community is to find more resources to connect them with services for their kids rather than us having to look so hard to find them on our own. That’s been our biggest experience and why I kind of get frustrated by the fact that I don’t think he is as far along as he could have been only because we never got the right connections to people or the right resources to get it done.

Even when good programs exist, knowledge of their presence does not always disperse in a way that would impact the entire community or families with autistic children; as a result, those in need may not benefit.

#### 3.2.3. Poor Patient-Provider Relationships

The patient-provider relationship is a crucial aspect of all health care encounters, including those focused on oral health. In this theme, participants described negative experiences with health care professionals (i.e., dental practitioners and clinic staff) that contributed to the strain of their patient-provider relationships.

Respect is key. First, caregivers expressed the desire for respectful exchanges with health professionals. As one parent stated, “…I think no matter what your income level is. No matter what your skin color is, you still should be treated with the same level of respect that everyone else is”. Unfortunately, participants described several unsatisfactory interactions with dental practitioners and clinic staff, which left an indelible mark impacting future experiences, regardless of the provider’s intention. For example, the following participant shared how a dentist rebuffed her parental expertise when she attempted to share information about her child, stating:

That dentist, he—even though I gave him the information [child’s autism diagnosis], he wasn’t quite receptive to it. He kind of like ignored it, and I kind of ended the session before he even went into her mouth… I guess it was more like the attitude towards it. It was more like, ‘I’ve been doing this for quite a while’. He kind of [gave] that impression like all kids were the same. And I’m like, ‘Yeah. This isn’t going to work out at all. Just because you have that mindset, this isn’t going to work for me. But you know what? Thank you’.

The dentist’s cavalier response resulted in the parent ending the session before treatment could begin, and the family never returned to that clinic. Another participant detailed an experience where the dentist made assumptions about the parent’s knowledge of their autistic child:

Like I remember going to [an appointment] and I was telling the doctor this happened and that happened… And [the practitioner] is like, ‘Oh, you know all that?’ and I’m thinking to myself, why wouldn’t I?… I was there every day. So, you know what I mean? [Practitioners] think, oh, [parents] don’t know much. And then when I start advocating effectively, not yelling and screaming, but understanding what our rights are because I have educated myself on that and taken advanced level advocacy training, I know how to advocate for my kid.

Sadly, this sentiment that some health care professionals exhibit bias in their patient-provider interactions is not uncommon among other participants. For example, in the following description, a caregiver shared how she felt that health professionals had shallow expectations of parents who identify as people of color regarding their level of involvement in their autistic child’s health pursuits; she stated:

I think sometimes when people of color go to dentists’ offices, or even doctors’ offices, there is this certain very low expectation in terms of what—you know, maybe the quality of health care [from the health care provider], or the [expected] level of engagement that the parent has with the child.

Flexibility of care. Several parents expressed understanding that working with their autistic child may present a unique set of experiences in the dental clinic, requiring flexibility and patience, which was not always provided by dental practitioners or their staff. One parent articulated how health professionals should treat autistic children, saying:

Just have patience. Autism is a spectrum, which means every child has a different level of tolerance, [and] level of ability. I think they just have to take their time. Then, explain everything. I think, for those [children with ASD] who are not as [verbal], I think a lot is shown in body language and facial expression. I think just embodying—treat them the way you want them to treat your child. Just have patience and explain everything. I think that’s pretty much it.

Another caregiver expressed their frustration that providers are not treating their child in the manner described above, succinctly stating, “I’m running out of patience with them if they’re running out of patience with [my child]”. As one parent explained, when one dental team refused to allow her autistic daughter to wear her headphones during the dental visit to minimize the sensory stimuli, the parent chose not to continue seeing that provider:

It was actually like the attitude of him and just the staff…[my child] walked in with her headphones because she does like what she’s comfortable with. And I explained that to him and the staff, and they were just like, ‘Okay, well, when the cleaning starts, she’s going to have to take that off’. I’m like, ‘No. She’s not taking it off. It’s not near her mouth. So, I’m not understanding why she has to take it off’. It was just like they had their own way of doing things. It’s like they had a routine and didn’t want to defer [sic] from it. So, I’m like, Yeah. This isn’t going to work out at all.

Sometimes the patient-provider relationship can be impacted by something as simple as the lack of eye contact during a visit or a less-than-desirable encounter with the front office staff. A parent detailed an experience with a new practitioner this way, “…it just seemed like when they were looking at his chart on the computer…and it just wasn’t as professional or as friendly as I think it should be”. Another caregiver shared how the entire team influences the overall patient-provider relationship while describing a visit to a dental clinic where the dentist was both indifferent to the parent’s concerns and impatient with the autistic child, and the front office staff was unfriendly. She said:

Because if you’re like that [indifferent and impatient] or your receptionist is like that [unfriendly], then I’m going to not want to come, or I’m feeling like, ‘Well, how are you going to treat my kid’? I’m just not going to like it. You’re providing a service, and I’m just not comfortable.

## 4. Discussion

In this study, African American parents identified barriers to the oral health of their autistic children, including difficulty maintaining good health practices in part related to child characteristics, lack of access to resources or financial burdens associated with the cost and amount of care, and negative clinic experiences with dental care professionals.

The first finding of this study highlighted that most parents actively sought positive health behaviors through the implementation of in-home oral health practices (i.e., brushing and flossing), despite the challenges they experienced. A common sentiment explicitly expressed by the parents was that they wanted better oral health experiences for their child. They were dedicated to complying with recommendations from their health care providers for best practices, in part related to the inconsistent oral care childhood experiences the parents described for themselves. Parents indicated they were willing to overcome their own dental fear and anxiety in order to make the best decision for their child. This finding is in line with previous work conducted with minoritized populations of autistic children that emphasizes how parents place the needs of their children at the forefront, and continually seek out better health care experiences for their children [14].

Caregivers also expressed some of the challenges and strategies they encountered when implementing good oral care practices with their autistic child. One parent stated that having an autistic child often means that seemingly mundane tasks like toothbrushing are not always routine and may require significant support. Therefore, creating effective routines may require additional tools and resources, such as modeling or seeking professional assistance. For example, utilizing siblings as models of desired behavior was one strategy that made brushing and flossing easier. This parallels what other researchers found regarding parents’ attempts to facilitate toothbrushing through modeling or familial assistance [22,32,40]. Similarly, occupational and behavioral therapists were identified as professionals who have been helpful in training and implementing daily oral care practices [41]. These are valuable considerations that may improve the ease with which these oral care related tasks can be accomplished, which can have lasting impacts beyond in-home oral health routines. It is possible that improving general oral health practices may lead to better experiences in a dental clinic which Corridore et al. (2020) shared in a systemtic review were greatly impacted by behavior and perceived patient cooperation [42].

The second finding of this study pertains to the lack of community resources, reduced health care access, and the financial burden associated with obtaining quality oral care as barriers to oral health, aligning with the current research [14,31,32]. Several participants shared that their communities were under-resourced and that even when programs existed, they remained unaware of them. There was a sense of disappointment that so much of the awareness of these programs resulted from word of mouth; they felt that better advertising strategies would make resources more accessible and more targeted to those in need, especially the autistic community. Some parents felt as though they were in pursuit of resources for their child and spent hours on the internet researching. This is inconsistent with literature suggesting that African American parents may have limited knowledge about risk factors or lower levels of health literacy—the capacity to obtain and understand health care information [13,43]. However, it does align with the previous finding of this study that parents are dedicated to provide the best care they can for their children.

One of the significant barriers to health care utilization described by caregivers is access to and type of insurance. Caregivers described the stress and fearfulness they felt because of the anticipation of the financial burden the cost of oral care may present, in addition to the other health care costs associated with their autistic child’s needs. Consequently, a leading strategy for reducing oral health disparities has been to increase access to insurance and discount dental plans. As such, most parents had some form of dental insurance for their child, regardless of their own insurance coverage status, which is similar to previous research that states access to public health insurance has increased significantly, specifically for ethnic minorities [44,45]. However, previous research indicated that this did not necessarily translate to increased dental service utilization for African Americans, but that did not hold true for this population [46,47]. A possible explanation in this study for finding high insurance and dental service utilization rates in families of autistic children with that they may have more familiarity with accessing health services and advocating for their child. This, along with parents’ desire to have better oral care experiences for their child, which was similar to the sentiment Latino caregivers expressed about prioritizing their child’s oral health, may be an essential facilitating factor that could be expanded upon to reduce oral health disparities [14].

The third finding spotlighted the significant impact patient-provider relationships have on oral health experiences, and how microaggressions and the intersection of race and autism diagnosis can negatively impact care for African American families. It is well-documented that African Americans have a tenuous history with the medical community and display evidence of mistrust for medical professionals [9,17,48]. This mistrust can lead to an avoidance of services and the perception of a lower quality of care, ultimately leading to increased oral health needs [49]. African American caregivers report that they are frequently dissatisfied with their child’s oral care and are more likely to say that their health practitioners do not spend enough time treating their child. This dissatisfaction may prevent them from seeking services [50]. Indeed, there was a general mistrust and belief among caregivers that the services recommended to them by dental practitioners were not required but instead were an attempt to upsell them (e.g., convince them to pay for services they do not need or pay for a more expensive service despite cheaper options).

Several participants also expressed how interactions with oral health professionals left a lasting negative impression. While none of the caregivers explicitly stated that they felt they were being discriminated against based on race or their child’s autism diagnosis, caregivers detailed encounters with dental health professionals where they were met with rigid treatment plans, felt disrespected or dismissed, and were confronted by implicit bias—which are all typical outcomes of institutionalized racism. These acts, regardless of intention, are microaggressions and act as a barrier to care. Previous research has indicated that even well-meaning, seasoned, dental care professionals who specialize in caring for pediatric autistic patients can act with unconscious bias and use “othering” language when referring to their autistic patients, which can stigmatize and further marginalize this population [51].

This study adds to the limited literature on the intersectionality of autism and race in African American families with autistic children by highlighting how different barriers can be compounded or doubled in amplitude. For example, caregivers discussed their stress at paying for both dental care and care related to their autistic child’s needs, and how they would manage those high costs. It can also be argued that African American patients experience microaggressions in healthcare settings due to established institutionalized and systemic racism; however, these caregivers also described receiving sub-optimal care due to their child being diagnosed as autistic. More work should be done to unpack this intersection of race and autism, including further exploring the relationship of both and their relationship to each other; this study is an important first step in this process of understanding the ways in which aspects of identity and social categories can shape experiences, opportunities, and participation in everyday living.

### Limitations

This was a qualitative study with a small sample size. Attempts were made to capture the population’s heterogeneity by including participants with different income levels and whose children were of different ages; this impacted the number of individuals representing each segment of the autistic population and further limited the ability to generalize the findings. However, this does not take away from the rich descriptions provided by the participants, which are best for understanding the factors and contexts of this understudied group. Therefore, future studies should examine the oral care experiences of African Americans with autistic children by including more participants to compare the child’s age, autism diagnosis, parent income or education, and other characteristics that may influence care. Lastly, incorporating other methods for evaluating oral care experiences and prolonged engagement with participants through a longitudinal design could be helpful to see how these processes unfold over time.

## 5. Conclusions

Due to the limited research that examines the intersection of autism, African American culture, and oral health practices, this study provides a rich picture of the barriers African American families with autistic children face when obtaining oral care. African American caregivers have demonstrated that despite their own negative dental experiences, they understand the value of good oral care practices and are willing to pursue care for their child. They need practitioners to promote a more inclusive environment, demonstrate patience and flexibility, and provide resources to aid in navigating health care costs. Strengthening patient-provider relationships is crucial to reducing oral health disparities, including improving levels of professionalism displayed by all staff in dental clinics, increasing understanding of implicit and explicit bias, and cultivating a communication style that is more widely received, acknowledging and embracing parental expertise. The ability of African American caregivers of autistic children to be resilient in the face of the many obstacles they encounter is a strength that should be supported. This study’s findings can inform the development of interventions and policies to improve oral health equity—the opportunity to attain the highest level of care notwithstanding social position.

## Figures and Tables

**Table 1:** Interview Participant Characteristics.

Descriptive Characteristics	N = 11 Interviewees (10 Children)
Interviewees	
Mother	10 (91%)
Father	1 (9%)
Child gender	
Female	2 (20%)
Male	8 (80%)
Child age range (years)	
4.0–7.11	3 (30%)
8.0–11.11	3 (30%)
12.0–14.11	4 (40%)
US Region	
West	2 (20%)
Midwest	1 (10%)
South	3 (30%)
Northeast	4 (40%)
SRS Severity	
Mild	3 (30%)
Moderate	2 (20%)
Severe	5 (50%)
Education	
<High School	
Some HS/Completed HS	2 (20%)
Some college/Associate’s degree	5 (50%)
Bachelor’s degree	2 (20%)
Graduate/Post Graduate degree	1 (10%)
Family Income	
Less than $20,000	3 (30%)
$20,000–34,999	1 (10%)
$35,000–49,999	3 (30%)
$50,000–74,999	1 (10%)
$75,000–99,999	1 (10%)
Over $100,000	1 (10%)

## Data Availability

Not applicable.
